# Early hyperreactive malarial splenomegaly and risk factors for evolution into the full-blown syndrome: a single-centre, retrospective, longitudinal study

**DOI:** 10.1186/s12936-015-1015-6

**Published:** 2015-12-02

**Authors:** Zeno Bisoffi, Stefania Leoni, Dora Buonfrate, Claudia Lodesani, Franklin Esoka Eseme, Geraldo Badona Monteiro, Stefania Marocco, Massimo Guerriero

**Affiliations:** Centre for Tropical Diseases, Sacro Cuore-Don Calabria Hospital, 37024 Negrar, Verona Italy; Medici senza Frontiere Italia, Via Magenta 5, 00186 Roma, Italy; Ospedale dell’Angelo, Via Don Federico Tosatto, 147, 30174 Venezia Mestre, Italy; Department of Computer Science, University of Verona, Strada Le Grazie 15, 37134 Verona, Italy

**Keywords:** Hyper-reactive malarial splenomegaly, Hyperreactive malarial splenomegaly, Tropical splenomegaly, Tropical splenomegaly syndrome, HMS, Early hyperreactive malarial splenomegaly, e-HMS, Chronic malaria, Malaria, *Plasmodium falciparum*

## Abstract

**Background:**

The hyperreactive malarial splenomegaly (HMS) represents a chronic, potentially fatal complication of malaria. Case definition includes: gross splenomegaly, high level of anti-malarial antibody and IgM, response to long-term anti-malarial prophylaxis. In this study, a large series of patients not fully meeting the case definition was tentatively classified as early hyperreactive malarial splenomegaly (e-HMS). The main research questions was: does “e-HMS” tend to evolve to the full-blown syndrome? And if so, what are the main factors influencing this evolution?

**Methods:**

Retrospective, longitudinal study. The patient database was searched to retrieve all potentially eligible patients. e-HMS was defined by splenomegaly of any size (with or without raised IgM), high anti-malarial antibody titre and exclusion of other causes of splenomegaly. The clinical outcome at following visits was analysed in relation to re-exposure to malaria, and to treatment (only part of the patients with e-HMS were treated with a single anti-malarial treatment and advised to follow an effective anti-malarial prophylaxis, if re-exposed). The association of the outcome with the main independent variables was first assessed with univariate analysis. A stepwise logistic regression model was then performed to study the association of the outcome with the main independent variables.

**Results:**

One hundred and twenty-six subjects with e-HMS were retrieved. Eighty-one had at least one follow-up visit. Of 46 re-exposed to malaria for a variable period, 21 (46 %) had progressed, including 10/46 (22 %) evolving to full-blown HMS, while of 29 patients not re-exposed, 24 (93 %) had improved or cured and five (7 %) progressed (p < 0.001). At logistic regression re-exposure was confirmed as a major risk factor of progression (OR 9.458, CI 1.767–50.616) while treatment at initial visit was protective (OR 0.187, CI 0.054–0.650).

**Conclusion:**

e-HMS should be regarded as a clinical condition predisposing to HMS. Although the case definition may include false positives, e-HMS should be treated just as the full-blown syndrome. A single anti-malarial treatment is probably adequate, followed by effective prophylaxis for patients exposed again to malaria transmission.

**Electronic supplementary material:**

The online version of this article (doi:10.1186/s12936-015-1015-6) contains supplementary material, which is available to authorized users.

## Background

The hyperreactive malarial splenomegaly (HMS), previously referred to as tropical splenomegaly or tropical splenomegaly syndrome (TSS) is a chronic complication of malaria. Patients have high levels of anti-malarial antibody [1, 2], as a result of the chronic antigenic stimulation, which seems to be an important factor in the development of the syndrome. Although the exact mechanism is uncertain, evidence suggests that repeated or chronic exposure to malaria elicits exaggerated stimulation of polyclonal B lymphocytes, leading to excessive and partially uncontrolled production of immunoglobulin M (IgM) as the initiating event [3]. IgM is polyclonal and there is no evidence to attribute the syndrome to any malaria species in particular [1], although *Plasmodium falciparum* is by and large the predominant species in the countries where the syndrome has been described [4]. A history of chronic splenic enlargement [5, 6] differentiates HMS from the splenomegaly observed in acute malaria.

At the very beginning patients might not have any symptoms. If HMS is not cured most patients report unspecific symptoms like asthenia, abdominal discomfort and other. Patients adapt physiologically to chronic anaemia, which develops due to hypersplenism [7, 8] and become symptomatic when it is severe. Hepatomegaly is also common. Main complications are represented by an increasing frequency of infectious diseases and haemolytic disorders, and mortality may exceed 50 % if the syndrome is left untreated [9]. In particular, especially pregnant women are susceptible to episodes of massive haemolysis, which are usually preceded by febrile episodes [10].

After excluding other known causes of splenomegaly, “tropical splenomegaly syndrome” (the former name of HMS) was defined as a separate entity and Fakunle set clear diagnostic criteria in 1981 [11], that were subsequently reviewed by Bates et al. [12]. The major diagnostic criteria include: massive splenomegaly, high anti-malarial antibody titre, high IgM value (local mean +2SD) and a clinical-immunological response to prolonged anti-malarial treatment or to a prophylactic regimen [13]. HMS has also been diagnosed in expatriates after a prolonged period of exposure in endemic areas [14].

At the centre for tropical diseases (CTD), in the last 25 years, a relevant number of cases have been observed, both in immigrants from malaria endemic areas and in Italian citizens who had lived for a prolonged period in such areas. Furthermore, over the years it has been also observed that some patients, who initially had only some of the case definition criteria of the HMS (in particular with light or moderate, instead of massive splenomegaly), once examined again after a further stay of variable duration in endemic areas, developed all the HMS criteria. It was, therefore, supposed that it is possible to identify and diagnose an early stage of HMS, here below defined as “early HMS” (e-HMS). As the latter was not considered in medical literature, these patients were usually not treated, unless malaria parasites were identified in blood (thus defining a chronic malarial infection). Once the early syndrome was hypothesized, a number of patients considered potentially affected were then treated for malaria and advised to follow an effective prophylaxis in case they had to go back to endemic areas.

The main objectives of this research were:

- to assess if e-HMS, that has not been so far considered as a nosological entity, tends to evolve to the full-blown HMS;
- if so, if the evolution of e-HMS is significantly different between patients re-exposed and not re-exposed to malaria transmission, and
- if the evolution of e-HMS is significantly different between patients who are treated for malaria versus those who are not.

## Methods

### Study design

Retrospective, longitudinal study aiming to identify patients with e-HMS observed from 1 January 1990 to 31 January 2014 and subsequently visited at follow-up (at least once after initial visit). The population included in the analysis had to be selected according to the following case definition of e-HMS:

- anti-malarial antibody titre (IFAT-Biomérieux) > 1/160, PLUS:
- splenomegaly (echographic longitudinal diameter ≥ 12 cm or palpable lower pole of the spleen) AND/OR
- high IgM level (≥2.5 g/L), AND
- no other identified causes of splenomegaly or of raised IgM, such as: schistosomiasis, HBV, HCV, HIV, brucellosis, leishmaniosis, autoimmune disease, cirrhosis, haemoglobinopathies, and other haematological conditions, such as leukemia, lymphoma, Waldenstrom disease.

Patients with splenomegaly or with raised IgM, observed during the study period, and without any of the mentioned alternative diagnoses, were retrieved from the CTD patient database as indicated below. Those with anti-malarial antibody titer ≥160, and/or with history of ≥3 years stay in malaria-endemic areas and reporting ≥10 acute malaria episodes, were initially included. Patients meeting the case definition of full-blown HMS as outlined above were excluded. The remaining subjects, meeting the tentative case definition of e-HMS, were retained for further analysis. The main epidemiological, clinical and laboratory findings were recorded at baseline. From baseline, patients were then traced prospectively for any further visit and, in case any follow-up contacts were recorded, follow up data was included in the study file. For all patients having at least a follow-up visit after initial observation, the last follow-up visit was considered for the outcome assessment in relation with re-exposure or not to malaria-endemic areas and to treatment or not at initial visit, as well as with the other main independent variables.

### Data source

CTD patient database (electronic since 1995, paper-based from 1990 to 1994). Echography of the upper abdomen, as well as immunoglobulin dosage and anti-malarial antibody testing, were part of the routine screening of patients with a history of a long stay (>1 year) in a malaria endemic country and reporting several malaria episodes.

### Data entry and data elaboration

Data was exported to a pre-structured Excel file (Additional file 1), including:

- (T1) baseline;
- (T2) short-term follow up visits (1–6 months after first observation);
- (T3) long-term follow up visits (≥6 months after first observation).

### Definition of main diagnostic criteria for e-HMS

- Splenomegaly: massive if bipolar diameter ≥ 16 cm or if lower pole beyond the umbilical line; moderate, ≥14 and <16 cm or lower pole palpable without inspiration; mild, ≥12 and <14 cm or lower pole palpable on inspiration only; normal spleen, <12 cm or lower pole not palpable.
- Anti-malarial titre: Indirect Fluorescent Antibody Test (IFAT, Biomérieux) titre ≥ 1:160. In the few cases of missing serology, a prolonged stay in an endemic area (≥3 years) and over 10 malarial episodes reported, was considered equivalent to a high anti-malarial antibody titer.
- Serum IgM level, two standard deviations above the local mean, corresponding to ≥2.5 g/L.

### Definition of follow-up criteria

- Cured: absence of splenomegaly and normal IgM titer;
- Improved: ≥0.5 cm decrease of the spleen diameter, or unchanged spleen diameter and ≥0.5 g/dL decrease of IgM level;
- Unchanged: <0.5 cm or no variation of the spleen diameter and <0.5 cm or no variation of IgM level;
- Progressed: ≥0.5 cm increase of the spleen diameter, or unchanged spleen diameter and ≥0.5 g/dL increase of IgM level;
- HMS: patients with e-HMS at initial visit, meeting the case definition of full-blown HMS (as resumed in Introduction) at follow-up.

### Laboratory methods for malaria detection

Patients were screened for *Plasmodium* using one of the following methods: QBC Malaria Test (QBC Diagnostics Inc, Philipsburg, US) or Malaria antigen Rapid Detection Test (RDT) (different commercial devices were used over time). For all positive results, confirmation with microscopy was also carried out.

### Statistical analysis

Data was analysed using the software STATA IC 14 (StataCorp, 4905 Lakeway, College Station, TX, 77845, USA) [15]. For categorical variables the absolute, relative and cumulate frequencies were calculated. For continuous variables, the median and IQ range were considered. The univariate association between categorical variables was evaluated using the χ^2^ test. The Fisher exact test was used if appropriated. A stepwise logistic regression model with forward selection was performed to study the association of the main dependent variable (outcome at follow-up) with the independent variables such as: sex; ethnicity; re-exposure to malaria; presence/absence of symptoms and of malaria parasites at first visit; treatment or not at first visit; age; time elapsed between the first observation and follow-up. Potential interaction among the variables was also studied with the same model. Significance values for entry in (pe)/removal from (pr) the model were set at 0.10 and 0.15, respectively.

### Ethical issues

Subjects were anonymously coded in the database, unlinked from any information identifying the source individuals. Although the study was retrospective and no action on patients was involved, the study protocol was nevertheless submitted to the Ethics Committee of the Coordinating Site (Comitato Etico Provinciale di Verona) for approval. The latter acknowledged the study protocol and formally authorized the study (protocol n. 43713 of 29th September, 2014).

## Results

### Study population

**Fig. 1:**
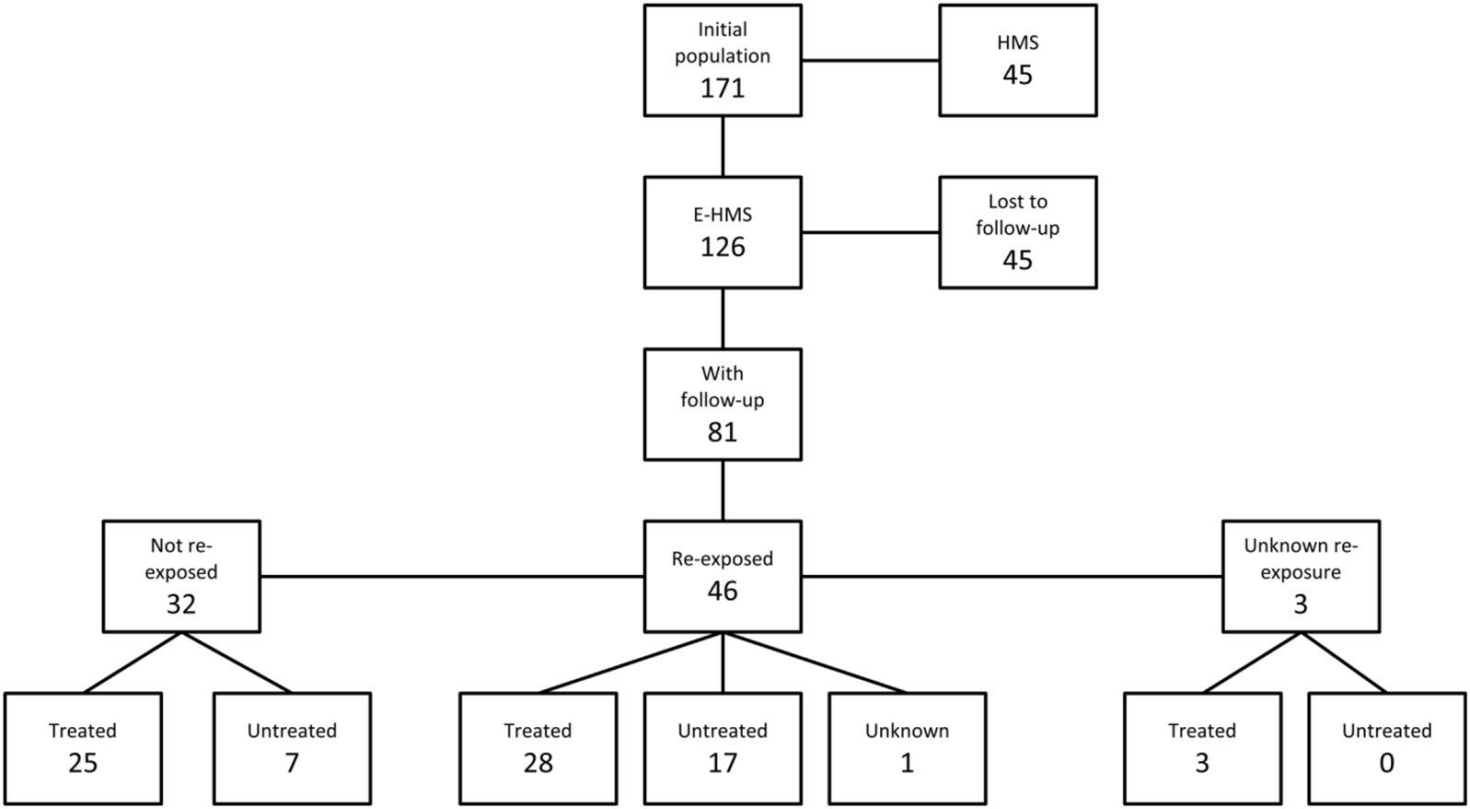
Study flow-chart

The patient flow is resumed in Fig. 1. From the initially retained sample of 171 patients, 45 were full-blown HMS. Among the 126 patients with e-HMS, 81 (64.3 %) had at least one follow up visit. Forty-six had been re-exposed to malaria transmission in endemic countries before follow-up, 32 had remained in Italy, and for three patients this information was missing. Of the 81 subjects with follow-up, 56 (70 %) were treated at initial visit (28 re-exposed, 25 not re-exposed and three with unknown re-exposure status), and 24 were not (17 re-exposed and 7 not re-exposed), while for one (re-exposed) this information was missing.

Most of the subjects included met the main case definition criteria as outlined in “Methods”. Anti-malarial antibodies were available for 86/97 Caucasian patients and for 20/29 non-Caucasians, while in the other cases the inclusion was based on the alternative criteria of ≥3 years stay in malaria-endemic areas and ≥10 acute malaria episodes. The median value of anti-malarial antibody titre was 2560 for Caucasians (IQ range 1280–5120) and 1280 for non-Caucasians (IQ range 320–5120).

### Baseline characteristics of the study population: patients with at least one follow-up visit versus lost to follow-up

Table 1 compares the main baseline characteristics between the e-HMS subjects who were followed-up and those who were lost. A substantial similarity is observed between the two subgroups.

**Table 1:** Baseline characteristics of subjects diagnosed with early HMS (e-HMS), with follow-up (Follow up) versus lost to follow up (Lost)

Characteristic	All (n = 126)	Follow up (n = 81)	Lost (n = 45)	p value
Age, years [median (IQ range)]	53.7 (38.9–61.4)	53.4 (38.9–60.9)	55.9 (41.5–62.7)	0.485
Gender (F/M)	52/74	31/50	21/24	0.359
Ethnicity (Caucasian/other)	97/29	62/19	35/10	0.875
Exposure, years [median (IQ range)]	20.0 (11.5–29.5)	20.0 (12.5–28.5)	19.5 (10.0–31.0)	0.839
Presence of symptoms, Y/N	114/10	74/6	40/4	0.742
Spleen diameter, cm [median (IQ range)]	14.0 (13.0–15. 0)	14.0 (13.0–15. 0)	13.5 (12.9–15.0)	0.083
Presence of hepatomegaly, Y/N	43/81	30/50	13/31	0.373
Hb, mg/dL [median (IQ range)]	12.4 (11.0–13.9)	12.3 (11.0–13.3)	12.75 (11.0–15.2)	0.264
RBC × 10^6^/µL [median (IQ range)]	4.2 (3.7–4.7)	4.1 (3.7–4.7)	4.5 (3.7–5.0)	0.233
Plt × 10^3^/µL [median (IQ range)]	173.0 (135.0–228.0)	170.0 (137.0–217.0)	179.0 (127.0–259.0)	0.367
ESR, mm/h [median (IQ range)]	26.0 (12.0–50.0)	25.0 (14.0–47.0)	28.0 (9.0–56.0)	0.892
IgM, g/L [median (IQ range)]	2.8 (1.9–3.7)	2.9 (1.9–3.8)	2.8 (1.9–3.6)	0.727
Plasmodium in blood, Y/N	36/51	26/33	10/18	0.460

### Baseline characteristic of subjects re-exposed vs not re-exposed to malaria transmission

Most subjects (46/78 or 59 %) were re-exposed to malaria transmission before follow-up (this data was missing for three patients). Table 2 summarizes the baseline characteristics of the two groups.

**Table 2:** Baseline characteristics of subjects diagnosed with early HMS (e-HMS), re exposed versus not re exposed to malaria transmission before follow up

Characteristic	All (n = 81)	Re-exposed (n = 46)	Not re-exposed (n = 32)	p value
Age, years [median (IQ range)]	53.4 (38.9–60.9)	54.0 (49.3–61.0)	37.5 (25.2–60.1)	0.003
Gender (F/M)	31/50	19/27	11/21	0.536
Ethnicity (Caucasian/other)	62/19	45/1	14/18	0.000
Exposure, years [median (IQ range)]	20.0 (12.5–28.5)	17.5 (9.5–29.0)	22.0 (16.5–27.5)	0.124
Presence of symptoms, Y/N	74/6	40/5	31/1	0.391
Spleen diameter, cm [median (IQ range)]	14.0 (13.0–15. 0)	14.0 (12.9–15.0)	15.0 (14.0–15.5)	0.011
Presence of hepatomegaly, Y/N	30/50	20/25	10/22	0.242
Hb, mg/dL [median (IQ range)]	12.3 (11.0–13.3)	12.3 (11.4–13.2)	12.3 (10.8–14.0)	0.870
RBC × 10^6^/µL [median (IQ range)]	4.1 (3.7–4.7)	4.1 (3.7–4.3)	4.6 (3.8–4.8)	0.042
Plt × 10^3^/µL [median (IQ range)]	170.0 (137.0–217.0)	169.0 (149.5–214.0)	183.0 (123.0–217.5)	0.674
ESR, mm/h [median (IQ range)]	25.0 (14.0–47.0)	25.0 (13.0–51.0)	23.5 (14.5–41.0)	0.905
IgM, g/L [median (IQ range)]	2.9 (1.9–3.8)	2.9 (2.1–4.7)	2.2 (1.6–3.6)	0.229
Plasmodium in blood, Y/N	26/33	14/16	11/15	0.743

Info on exposure missing in three cases

The median age of re-exposed subjects was higher: 54 years versus 37.5 years (p = 0.003). Most patients re-exposed were Caucasian (45 vs one, p < 0.001). There was no significant gender difference. The average duration of exposure to malaria before observation was similar and so were most clinical and laboratory findings. The median diameter of the spleen was higher in not re-exposed subjects (15 vs 14 cm, p = 0.011) and so was the median RBC count (4.6 vs 4.1 × 10^6^/µL, p = 0.042), but the Hb value was similar in the two groups.

### Baseline characteristics of e-HMS group, treated vs untreated

The characteristics of the e-HMS subjects who received/did not receive an anti-malarial treatment are summarized in Table 3. The two groups were similar for the variables considered, but for the presence of malaria parasites in blood (26 vs 26 in the treated group, 7 vs 0 in the untreated group, p = 0.014) and for the median Plt count, (158 vs 201 × 10^3^/µL, p = 0.014).

**Table 3:** Baseline characteristics of subjects diagnosed with early HMS (e-HMS), with follow-up: treated versus untreated

Characteristic	All (n = 81)^a^	Treated (n = 56)	Not treated (n = 24)	p value
Age, years [median (IQ range)]	53.4 (38.9–60.9)	54.0 (36.09–60.9)	49.8 (42.5–58.2)	0.502
Gender (F/M)	31/50	22/34	9/15	0.881
Ethnicity (Caucasian/other)	62/19	42/14	19/5	0.688
Exposure, years [median (IQ range)]	20.0 (12.5–28.5)	20.0 (13.0–28.5)	19.0 (13.0–31.5)	0.677
Presence of symptoms, Y/N	74/6	54/2	20/4	0.063
Spleen diameter, cm [median (IQ range)]	14.0 (13.0–15. 0)	14.0 (13.3–15. 0)	14.4 (13.0–15.0)	0.601
Presence of hepatomegaly, Y/N	30/50	20/36	10/14	0.614
Hb, mg/dL [median (IQ range)]	12.3 (11.0–13.3)	12.3 (10.7–13.3)	12.4 (11.9–13.9)	0.154
RBC × 10^6^/µL [median (IQ range)]	4.1 (3.7–4.7)	4.1 (3.7–4.6)	4.4 (4.0–4.7)	0.227
Plt × 10^3^/µL [median (IQ range)]	170.0 (137.0–217.0)	158.0 (129.0–208.0)	201.0 (165.5–250.0)	0.014
ESR, mm/h [median (IQ range)]	25.0 (14.0–47.0)	26.5 (14.5–42.0)	23.0 (16.5–41.5)	0.319
IgM, g/L [median (IQ range)]	2.9 (1.9–3.8)	2.9 (1.7–4.3)	2.8 (2.1–4.1)	0.693
Plasmodium in blood, Y/N	26/33	26/26	7/0	0.014

^a^Info on treatment missing in one case

### Symptoms

Most of the patients were symptomatic at first observation. Asthenia was the most frequently reported symptom, present in 44/97 Caucasians (45 %) and in 7/29 non-Caucasians (24 %). Fever (most often low-grade) was present in 37/97 Caucasians (38 %) and in 8/29 non-Caucasians (28 %).

### Parasite detection

Overall, *Plasmodium* was searched in 87 subjects out of 126 (59.2 %), with a positive result (usually at low parasite density) in 36/87 (41.4 %). *P. falciparum* was the species involved in all cases, but for one case of *Plasmodium malariae* and one of *Plasmodium vivax*, both in Caucasians. Among Caucasians, positive results were 27/67 overall, of which 3 (11 %) had only a positive RDT or QBC (with negative blood film), and of the remaining 24, 12 (50 %) had a parasite density <1000/µL (of which 3 with <50/µL). The highest value of parasite density was 10,500/µL, roughly corresponding to 0.25 %. Among non-Caucasians, positive results were 9/20 overall (45 %), of which 2 (22 %) had only a positive RDT or QBC, and of the remaining 7, 5 (71 %) had a parasite density <1000/µL (of which 2 with density <50/µL). The highest value of parasite density was 21,500/µL, roughly corresponding to 0.5 %.

### Outcome at follow-up of patients with e-HMS re-exposed versus not re-exposed to malaria transmission

The results of univariate analysis are reported in Table 4. If unexposed, the majority improved (20/32 = 62.5 %) or even healed (7/32 = 21.9 %), and none progressed, while almost half of subjects re-exposed progressed (11/46 = 23.9 %) or evolved to full-blown HMS (10/46 = 21.7 %) (p < 0.001).

**Table 4:** Outcome at follow-up of patients with e-HMS, re-exposed versus not re-exposed to malaria transmission

Outcome	Cured	%	Improved	%	Unchanged	%	Progressed	%	HMS	%	Tot
Re-exposed	13	28.3	12	26.1	0	0	11	23.9	10	21.7	46
Not re-exposed	7	21.9	20	62.5	5	15.6	0	0	0	0	32
Tot	20	25.6	32	41.0	5	6.4	11	14.1	10	12.8	78

p < 0.001, Fisher’s exact

### Outcome of early-HMS patients treated versus not treated for malaria at initial visit

The results are reported in Table 5. Most of the 56 treated patients healed (15/56 = 26.8 %) or improved (29/56 = 51.8 %), while almost half of the untreated patients progressed (5/24 = 20.8 %), or evolved to the full-blown-HMS (6/24 = 25.0 %) (p = 0.040). The final model of stepwise logistic regression was based on 77 observations and resulted globally statistically significant (p = 0.0004). The goodness of fit was modest (pseudo R^2^ = 0.2137) suggesting the need of confirming the results with a higher number of observations and/or with the association of other possible predictors, if any. Variables significantly associated with outcome in the final model (Table 6) were treatment, presence of asthenia and re-exposure to malaria-endemic areas, while age was close to significance. Re-exposure (OR 9.458, p = 0.009) and presence of asthenia at initial observation (OR 4.188, p = 0.035) were independently associated with progressed outcome, while treatment resulted associated with improved outcome (OR 0.187, p = 0.008).

**Table 5:** Outcome at follow-up of patients with e-HMS, treated versus not treated at initial observation

Outcome	Cured	%	Improved	%	Unchanged	%	Progressed	%	HMS	%	Tot
Treated	15	26.8	29	51.8	3	5.4	5	8.9	4	7.1	56
Not treated	5	20.8	6	25.0	2	8.3	5	20.8	6	25.0	24
Tot	20	25.0	35	43.8	5	6.3	10	12.5	10	12.5	80

p = 0.040, Fisher’s exact

**Table 6:** Summary results of stepwise logistic regression

Variable	Odds ratio (95 % conf. interval)	p value
Treatment at initial visit	0.1865272 (0.0535228–0.6500476)	0.008
Age	0.9510769 (0.9045759–0.9999683)	0.050
Presence of asthenia at initial visit	4.187701 (1.105679–15.8607)	0.035
Re-exposure to malaria-endemic areas before follow-up	9.457626 (1.767157–50.61614)	0.009
Constant	2.230569 (0.3019927–16.47536)	0.432

Odds ratio (95 % conf. interval) of the risk of worsening outcome for the independent variables retained by the model and respective p values

## Discussion

### Baseline characteristics of the study population

As 1/3rd of the study subjects were lost at follow-up, it was first assessed if this group was similar for the main baseline characteristics to the group of subjects with follow-up, which was indeed the case (Table 1). The same comparison was subsequently performed for the two main independent variables that were hypothesized to be associated with the final outcome: re-exposure or not to malaria and treatment at the initial visit. Here again, the two groups were substantially similar (Table 2), with a few important exceptions. The median age of the re-exposed group was significantly higher which was clearly correlated with ethnicity, as almost all re-exposed subjects were Caucasians, mostly missionary people who returned to the African country of residence, while more than half of the not re-exposed group was composed of immigrants who had come to Italy to stay. The difference in spleen diameter may find a logical explanation (it is possible that subjects with a bigger spleen were more frequently advised to avoid re-exposure) or be simply due to chance, similarly to the difference in RBC count (but not in Hb value). Treated versus untreated subjects (Table 3) were also similar, but for the presence of detectable *Plasmodium* in blood and for the platelet count (the two variable are correlated, as malaria is known to cause a transient drop in platelet count). Patients were more likely to be treated if malaria parasites were found in blood, which is by no mean unexpected. All potentially confounding variables were included in the log regression model.

### Outcome analysis

This retrospective, longitudinal study aimed to describe the clinical evolution of subjects presenting a history of long term exposure to malaria transmission and a splenomegaly (any spleen size), with or without a raised IgM level in blood. After excluding other possible causes of splenomegaly, a tentative definition of e-HMS was devised, as described in the “Methods”.

It has been reported that the development of HMS can be influenced by various aspects, including genetic predisposition [16–18], but the evaluation of these aspects was not a purpose of this study.

Returning to an endemic area for malaria proved to be a risk factor for the evolution of the syndrome. Almost half of all the subjects re-exposed to malaria progressed, versus none of those who remained in Italy until follow-up. Even more importantly, almost a quarter of the re-exposed group evolved to a full-blown HMS. Therefore, although the case definition of e-HMS may not be specific and may include patients with other (albeit unrecognized) causes of splenomegaly, the risk of evolution to HMS, a severe disease with high mortality, justifies in itself the recognition of the early stage of the disease, that should be properly treated. Treatment appeared to be independently protective against evolution of e-HMS to full-blown HMS.

### Study limitations

The main limitations of this study are inherent to the retrospective study design, in particular:

- loss at follow-up of several patients: the follow-up data was obtained retrospectively from the patients returned (often occasionally) to the observation after the first contact; anyway for about 2/3rd of the study subjects follow-up data was available, moreover as mentioned above the baseline characteristics of the two groups were in all similar;
- not uniform follow-up time, for the same reasons outlined above; however, this variable was duly included in the log regression model;
- differences between the two main groups of re-exposed/not re-exposed and of treated/not treated for some characteristics as discussed above;
- on logical grounds, for treatment at initial observation to be really effective, long term prophylaxis and/or frequent/intermittent anti-malarial treatment are also necessary [19, 20]. Unfortunately, while the subjects who were treated were of course also recommended to follow an effective prophylaxis if re-exposed, self-reported data on compliance with this recommendation proved not to be reliable and it was not possible to include this variable in the model.

However, at multivariate analysis including most potential predictors, the main findings (the tendency of e-HMS to evolve in subjects re-exposed to malaria, and in those not treated) was robust. Asthenia was the only symptom seemingly influencing the risk of evolution, although it cannot be excluded a chance association.

## Conclusions

It’s possible to identify an early stage of HMS, which has been defined as e-HMS, that tends to evolve into a full-blown HMS if malaria antigenic stimulus persists and if subjects are not treated for malaria. e-HMS is characterized by a spleen size ≥12 cm, even in the absence of high values of IgM level, or by raised IgM without splenomegaly, in subjects with a history of long term exposure to malaria transmission, and with high titre of anti-malarial antibodies. The case definition of the syndrome should be updated in order to include criteria to diagnose e-HMS, that should be recognized and treated. Although the diagnostic criteria of e-HMS may not be highly specific and include false positive subjects (with other causes of splenomegaly), treatment is warranted even if diagnostic certitude is lacking, considering the risk of evolution to HMS and its complications.

A randomized controlled trial would be the best way to rigorously assess the efficacy of initial anti-malarial treatment, followed by prophylaxis or possibly by intermittent anti-malarial treatment in case of re-exposure, on the evolution e-HMS. However, undertaking a similar trial may prove difficult in the field, also considering the necessarily long follow-up time. On the other hand, a multicentre retrospective study on a larger series of patients observed in non-endemic countries could corroborate the findings of this research and lay a more robust basis for its conclusions.

## Additional file

10.1186/s12936-015-1015-6 Study database.
